# Nosocomial Myiasis Caused by *Lucilia sericata* (Diptera: Calliphoridae) in a Pediatric Patient in Mexico

**DOI:** 10.1155/2020/1285459

**Published:** 2020-01-29

**Authors:** Hugo Martínez-Rojano, Herón Huerta, Luis M. Hernández-Triana, Eduardo Francisco Ruiz Pérez, Reyna Sámano

**Affiliations:** ^1^Departamento de Posgrado e Investigación, Escuela Superior de Medicina del Instituto Politécnico Nacional, Plan de San Luis y Díaz Mirón S/N, Colonia Casco de Santo Tomas, Delegación Miguel Hidalgo C.P. 11340, Mexico City, Mexico; ^2^Laboratorio de Entomología, Instituto de Diagnóstico y Referencia Epidemiológicos InDRE, Francisco de P. Miranda No. 177, Colonia Unidad Lomas de Plateros, C.P. 01480, Mexico City, Mexico; ^3^Animal and Plant Health Agency, Woodham Lane, Addlestone, Surrey KT15 3NB, UK; ^4^Terapia Intensiva del Hospital de Especialidades Del Niño y La Mujer “Dr. Felipe Núñez Lara”, Avenida Luis M, Av. Luis Vega Monroy 1000, Colinas del Cimatario, C.P. 76090, Santiago de Querétaro, Qro., Mexico; ^5^Departamento de Nutrición y Bioprogramación, Instituto Nacional de Perinatología, Secretaría de Salud Montes Urales 800, Lomas de Virreyes, Alcaldía Miguel Hidalgo, C.P. 11000, Mexico City, Mexico

## Abstract

Prevention of nosocomial myiasis, or hospital-acquired larvae infestation, should be an essential part of all hospital infection control programs. However, little is known about nosocomial myiasis, despite the extensive medical and psychological effects it has on patients and their families and the negative effects it has on hospitals' reputation and finances. This report describes a case of nosocomial myiasis of a 13-year-old boy who was admitted to a pediatric intensive care unit for congestive heart failure, anemia, uremic encephalopathy, hypertension, and severe respiratory distress. Ten days after admission, the pediatrician and the nurse perceived an increase in the volume of the gingival mucosa of the upper buccal vestibule and the presence of fly larvae. The maggots were sent to the Instituto de Diagnostico y Referencia Epidemiologicos for identification and were found to be *Lucilia sericata* larvae. This report highlights the need to educate medical and paramedical personnel, as well as creation and implement protocols in hospitals to avoid nosocomial myiasis and improvement of general sanitation.

## 1. Introduction

Nosocomial myiasis is an infestation of larvae during or after hospitalization that was not present or incubating at the time of hospital admission [1, 2]. Of the various types of myiasis, only secondary (accidental or facultative) myiasis can be nosocomial [3]. As nosocomial myiasis is a strong indicator of negligence in medical care, there is evidence that hospitals underreport this type of infestation to avoid associated legal and political implications [4]. Despite nosocomial myiasis' extensive medical and psychological impact on patients and adverse effects on hospitals' reputation and finances, guidelines for the prevention of nosocomial infections typically focuses on bacterial infections. These guidelines do not include the prevention of arthropod infestation, especially infestations by common flies that can easily pass into intensive care units (ICUs) [5]. We report a case of oral nosocomial myiasis caused by *Lucilla sericata* (*L*. *sericata*) in a pediatric patient in a pediatric intensive care unit (PICU) in order to share and explain the context surrounding this case. This case report is the first evidence of this species in a pediatric patient in Mexico, although this species is probably responsible for most reported cases of nosocomial myiasis [6].

## 2. Case Report

The patient described in this case is a 13-year-old boy originally from the state of Guanajuato, residing in the state of Queretaro, two neighboring states in central Mexico. The boy was raised in a lower-middle-class household and environment. The patient had a history of chronic renal failure controlled with hemodialysis. On April 8, 2019, the patient presented with severe respiratory distress, seizures, and generalized edema and was taken to a private hospital where he began treatment with mechanical ventilation, anticonvulsants, and hemodialysis. Once stabilized, he was immediately referred to a public specialty hospital and entered their Pediatric Emergency Department in critical condition on April 9, 2019. Once there, due to uremic encephalopathy, pneumonia, and the need for mechanical ventilation, the patient was transferred to the hospital's PICU.

**Figure 1:**
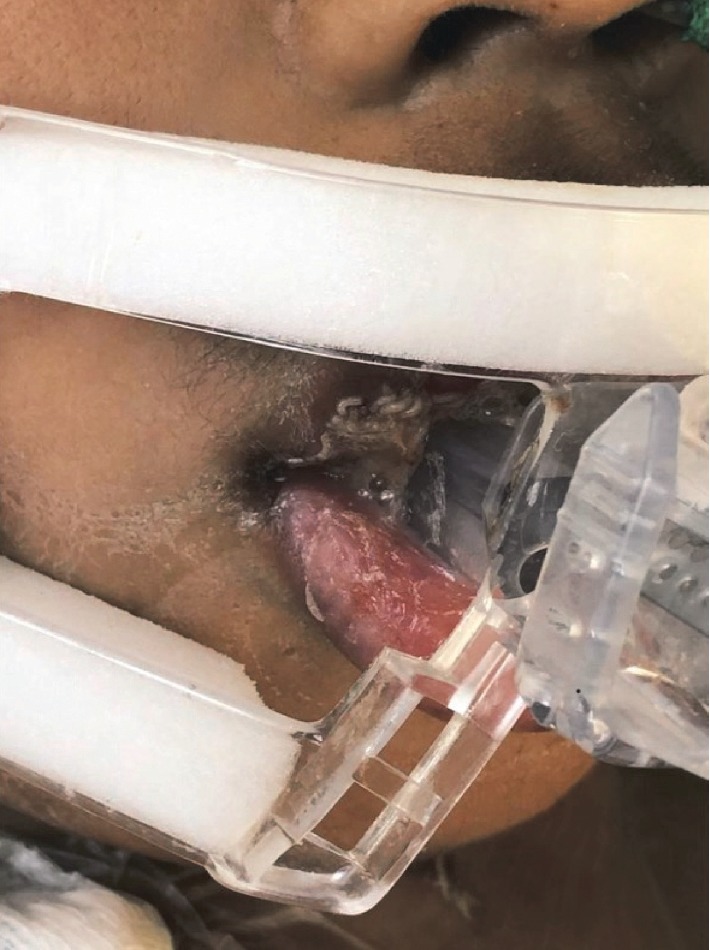
Patient's with nosocomial myiasis. Presence of larvae between upper lip and tongue.

Ten days after the patient's admission to the PICU, the pediatrician and the nurse observed an increase in the volume of the upper lip, with secretion and release of numerous gingival mucosa larvae from the anterior and superior buccal vestibule (Figure 1). The pediatrician immediately performed an intraoral examination, where he observed deficient hygiene and generalized periodontal disease with numerous cavities and halitosis. The maggots were removed, and the oral cavity was cleaned with sodium bicarbonate mouthwashes daily for eleven days. The infestation was treated with ivermectin at a dose of 0.2 mg/kg/d, orally for five days, which paralyzed and killed the larvae, allowing the mouth lesions to heal. Because of the severity and evolution of the patient's chronic renal failure, he then had to undergo a gastrostomy and tracheotomy (unrelated to the myiasis). After these successful surgeries, he was discharged 21 days later to the nephrology department to continued with his renal replacement therapy.

The larvae collected were sent to the entomology laboratory of the Instituto de Diagnóstico y Referencia Epidemiológicos (Epidemiological Diagnostic and Reference Institute or InDRE) for taxonomic analysis. The specimens collected were of 1.6–2.0 mm (mean of 1.8 mm) in length and of a beige color. The larvae were initially stored for two days in formaldehyde and subsequently preserved in 70% ethyl alcohol permanently. Three larvae were prepared for microscopic study by first performing a prerinse with 8% potassium hydroxide and then rinsing them with 10% glacial acetic acid, isopropyl alcohol, clove oil and finally preserving them in the Euparal® assembly medium (BioQuip Products, Inc. USA) on a microscope slide [7]. Photographs were taken of the slides using an Infinity 1 Lumenera® digital camera through Olympus microscopes (BX50 and SZX7) and then edited in Adobe Photoshop®.

**Figure 2:**
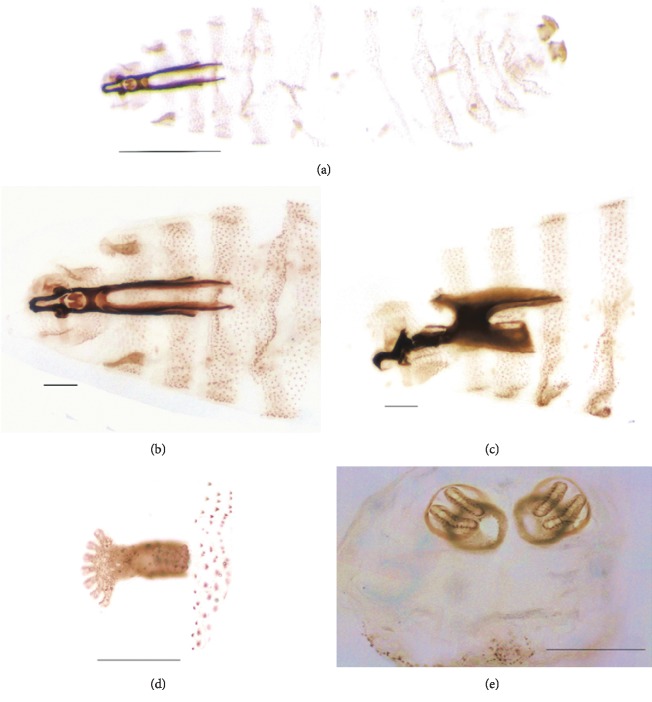
*Lucilia sericata*. Second stage larva. (a) Specimen, dorsal view (a) = 2.5x. (b) Cephaloskeleton, dorsal view. (c) Cephaloskeleton, lateral view ((b and c) = 4x). (d) Anterior stigma. (e) Posterior stigmata plates ((d and e) = 10x). Scales: (a) = 0.5 mm; (b)–(e) = 0.1 mm.

The entomology laboratory at the InDRE used these slides (magnified to 4x and 10x) and a SZX7 stereoscope and an Olympus BX43 optical microscope to identify the species of the larvae using their morphological characteristics. A literature review was performed to inform the taxonomical differentiation of these specimens against other members of the Oestroidea superfamily, especially the Calliphoridae, Cuterebridae, and Oestridae families. The characteristics that differentiated Calliphoridae family from other families were the following: body cylindrical and tapering; cephaloskeleton without long window in dorsal cornua and with developed parastomal bar; anterior spinose bands on all thoracic and most abdominal segments fully developed; posterior spiracles never in deep spiracular cavity or on long stalks, around spiracular field seven pairs of papillae; slits of posterior spiracles linear [8]. The main characteristics used to classify the species of the larvae were its spiracles, peritrema, peritrema opening, tubers, mouth hooks, respiratory tracheas, cephalopharyngeal skeleton, and the number of segments (Figure 2). The morphological identification revealed that the larvae obtained from the patient belonged to the Calliphoridae family, and all were in larval stage II of their life cycle [9].

In order to molecularly support our morphological identification, we employed the COI DNA barcoding approach as detailed in Hebert et al. [10]. DNA extraction was performed using a modified Hotshot technique as in Hernández-Triana et al. [11]. In short, two whole larvae were placed directly into 50 *μ*L of alkaline lysis buffer in a 96-well plate and then sonicated in a water bath for 15 min. The plate was subsequently incubated in a thermocycler for 30 min at 94°C and cooled for 5 min at 4°C, after which 50 *μ*L of neutralizing buffer was added to each sample ensuring that both buffers mix well during this step. The lysate was stored at −80°C until analysis, while the larvae were preserved in ethanol 99%. For amplification of the *COI* DNA barcoding region, we used the PCR forward forward primer LCO1490 (5′-GGT CAA CAA ATC ATA AAG ATA TTG G-3′) and reverse primer HCO 2198 (5′-TAA ACT TCA GGG TGA CCA AAA AAT CA-3′) which amplify the 658-bp target region of the *COI* gene [10, 11]. PCR products were obtained using the following reaction mix in a final volume 50 *μ*L: 2 *μ*L of DNA template, 25 *μ*L of H_2_O, 5 *μ*L of NH_4_, 5 *μ*L of dNTPs (2 pmol/*μ*L), 2.5 *μ*L of MgCl_2_ (25 pmol), 0.1 *μ*l of Bioline Taq Polymerase (Bioline Ltd), 5 *μ*L of each forward and reverse primers (each at 10 pmol/*μ*L), and 0.38 *μ*L of bovine serum albumin (20 mg/mL). The thermal profile consisted of an initial denaturation step at 94°C for 1 min, 5 cycles of preamplification of 94°C for 1 min, 45°C for 1.5 min, 72°C for 1.5 min, followed by 35 cycles of amplification: 94°C for 1 min, 57°C for 1.5 min and 72°C for 1 min, followed by a final elongation step of 72°C for 5 min. Paired bidirectional sequence traces were combined to produce a single consensus sequence for each specimen. Blast searchers were carried out in NCBI and BOLD public databases. The sequences for both specimens matched 100% to *L*. *sericata* (accession number KT72854).

## 3. Discussion

We report this case of nosocomial myiasis because of our absolute certainty that this infestation was acquired in the PICU; we reached this diagnosis because of the length of this patient's stay coupled with the *L*. *sericata* life cycle. *L*. *sericata* life cycle lasts an average of 456 hours from the time the female fly deposits the eggs to the adult phase (eggs: 24 hours; larval stages I, II, III: 264 hours; prepupa: 120 hours; and pupa: 120 hours) at a temperature of 23.2°C [12]. *L*. *sericata* typically take 12 days to reach the end of the larval stage at room temperature, but this duration may be shorter, because at higher temperatures, the time is shorter, and the patient had been hospitalized for 10 days when the stage II larvae were found.

The larvae of *L*. *sericata* have been the most frequently reported causes of cutaneous, traumatic, ophthalmic, aural, nasal, enteric, urogenital, and nosocomial myiasis in Poland, Serbia, Turkey, Iran, United States, Czech Republic, Canada, Jamaica, Honduras, India, French Guiana, New Zealand, Japan, and the Republic of Korea [1, 4, 6, 13, 14]. Including this publication, five cases of human myiasis have been reported in Mexico of which three correspond to nosocomial myiasis. *L*. *sericata* is the causative agent of two of the three reported cases of nosocomial myiasis [15, 16], similar to that reported in the United States, Iran, Czech Republic, and Turkey [17–19], where the causative agent of nosocomial myiasis was *L*. *sericata*.

Nosocomial myiasis occurs in immobile, weakened, seriously ill, semiconscious, or unconscious patients with multiple risk factors, such as the presence of trauma, surgical or puncture wounds, abscess drainage, assisted breathing and tracheal tubes, or injuries secondary to continuous exposure of mucous membranes [6, 14]. Our patient had several known risk factors: he was unconscious and intubated, mechanically ventilated, and had poor oral hygiene, which were the same risk factors reported in patients in the United States and in the two patients previously reported by our research group [15]. Our case is the third report of nosocomial myiasis in Mexico and the first of a pediatric patient hospitalized in PICU. Our case is similar to myiasis reported in developed countries detected in hospitalized patients in the ICU [14, 20–23], which is an area of restricted access and with rigorous sanitary control measures designed to avoid nosocomial infections or infestations. This is different from reports from lesser developed regions (namely, Iran, Turkey, India, and the Czech Republic) where cases of myiasis generally present in the area of emergency and hospitalization where sanitary control measures are not as strict as in ICUs [21].

If oral myiasis is diagnosed at the initial stage of the infestation, it can be benign and asymptomatic. Fortunately, in the case we report here, the diagnosis was made in the initial phase of the infestation, the myiasis was appropriately treated, and the patient responded to the treatment adequately, with no lasting complications. However, a delayed diagnosis can cause severe problems, namely, if the diagnosis occurs after the larvae have penetrated into tissue [6]. Tissue damage is particularly critical for severe or debilitated patients.

The usefulness of the analysis of the genetic variation of DNA to obtain reliable information about the larval species has been pointed out. To solve this problem, the specific information contained in the DNA molecule that is characterized by its immutability during all phases of the fly's life cycle, its presence in any of its phases and high stability, which facilitates taxonomic identification at any stage of the life cycle, independent of preservation methods [24, 25]. Consequently, the techniques based on the analysis of mitochondrial DNA have numerous advantages over morphological identification when specimens are damaged or lack the necessary characteristics for specific morphological classification, as was the case here [26].

No matter how benign the end result, nosocomial myiasis has a psychological impact on the patient and family members and seriously damages the reputation and financial capacity of the hospital [6]. This case was no exception, and its psychological impact on this particular patient's family was very important. Nosocomial infestations may also imply broader negligence on the part of the hospital authorities and possible failures in the sanitary and structural conditions of the hospital. This negligence could be grounds for legal action, involving significant losses for hospitals if it is sued for negligence [27, 28]. In our case, this was the first case of nosocomial myiasis at a specialty hospital. Due to the hospital staff's swift and transparent response, coupled with effective treatment, the relatives did not file any complaints against the institution.

The outbreak dengue in Kandy Teaching Hospital, Sri Lanka [29], that of nasal myiasis in an intensive care unit linked to the infestation of mice [30], and the present case, reminds us the importance of always paying attention to general sanitation of a hospital premises and keeping those free from harmful insects, especially elimination of potential breeding places, being vigilant of such infections/infestations and taking prompt action when such a nosocomial infection occurs.

In addition to treating this particular case of myiasis, preventive measures increased number of nurses, and a new protocol were implemented to avoid new cases. For instance, the protocol in order to control the fly population considered keeping garbage always covered and the use of nets for windows, among others. We report this case as a positive example of early diagnostic, notification, identification, and prevention of future myiasis. Our group was privileged in the sense that Mexico has a network of public health laboratories that could perform an entomological diagnosis of the larvae, as well as a network of epidemiologists facilitating nosocomial infection/infestation reporting and surveillance. We recommend that in general, myiasis prevention should be given greater importance by hospital authorities, especially in hospital units with structural and sanitary deficiencies. Hospitals must adopt suggested preventive measures to reduce the incidence of nosocomial myiasis, minimize fly populations associated with myiasis in humans, and promote strategies to respond to nosocomial myiasis [14, 30]. A prerequisite for the presence of nosocomial myiasis is the lack of awareness among staff who, although they know that flies are not hygienic and carry diseases, do not realize that they can also cause nosocomial myiasis. Education and general sanitary measures are a cornerstone in the strategy for nosocomial myiasis prevention.
